# A quantum material spintronic resonator

**DOI:** 10.1038/s41598-021-93404-4

**Published:** 2021-07-23

**Authors:** Jun-Wen Xu, Yizhang Chen, Nicolás M. Vargas, Pavel Salev, Pavel N. Lapa, Juan Trastoy, Julie Grollier, Ivan K. Schuller, Andrew D. Kent

**Affiliations:** 1 Department of Physics, Center for Quantum Phenomena, New York University, New York, NY 10003 USA; 2 Department of Physics, Center for Advanced Nanoscience, University of California-San Diego, La Jolla, CA 92093 USA; 3 Unité Mixte de Physique, CNRS, Thales, Université Paris-Saclay, 91767 Palaiseau, France

**Keywords:** Spintronics, Ferromagnetism

## Abstract

In a spintronic resonator a radio-frequency signal excites spin dynamics that can be detected by the spin-diode effect. Such resonators are generally based on ferromagnetic metals and their responses to spin torques. New and richer functionalities can potentially be achieved with quantum materials, specifically with transition metal oxides that have phase transitions that can endow a spintronic resonator with hysteresis and memory. Here we present the spin torque ferromagnetic resonance characteristics of a hybrid metal-insulator-transition oxide/ ferromagnetic metal nanoconstriction. Our samples incorporate ${\mathrm {V}}_2{\mathrm {O}}_3$, with Ni, Permalloy (${\hbox {Ni}}_{80}{\hbox {Fe}}_{20}$) and Pt layers patterned into a nanoconstriction geometry. The first order phase transition in ${\mathrm {V}}_2{\mathrm {O}}_3$ is shown to lead to systematic changes in the resonance response and hysteretic current control of the ferromagnetic resonance frequency. Further, the output signal can be systematically varied by locally changing the state of the ${\mathrm {V}}_2{\mathrm {O}}_3$ with a dc current. These results demonstrate new spintronic resonator functionalities of interest for neuromorphic computing.

## Introduction

Novel magnetic and ferromagnetic resonance characteristics can be achieved at interfaces by coupling quantum materials and metallic ferromagnets^1^. For example, the first-order structural phase transition of transition metal oxides has been shown to drastically influence the coercive field of a coupled Ni layer^2–4^. Further, ferromagnetic resonance frequencies of metallic ferromagnets have also been shown to be influenced by proximal transition metal oxides^5,6^. These advances open new possibilities for spintronic devices, which thus far have almost exclusively relied on the characteristics of metallic ferromagnets^7^. Here we demonstrate this promise for nanoconstriction type spintronic resonator.

In spintronic resonators spin-polarized currents excite spin dynamics. In nanoconstriction type devices^8^ the spin-Hall effect in a heavy metal produces a spin current that is transverse to the charge current^9^. A charge current in a heavy metal layer thus leads to spin currents that can exert a torque on the magnetization of an adjacent ferromagnetic layer^10,11^. The torque and associated magnetic excitations are typically concentrated in the region with the highest current density, the lithographically defined nanoconstriction. The magnetic excitations lead to variations in the nanoconstriction’s resistance that can be detected electrically. Such spintronic resonators are nonlinear, and tunable in phase, amplitude and frequency^12,13^. This makes them of great interest in neuromorphic computing where their response to external perturbations, phase locking and mutual synchronization can be exploited^14–17^.

In this article we show that several new spintronic resonator functionalities emerge in nanoconstrictions formed from a ferromagnet/${\mathrm {V}}_2{\mathrm {O}}_3$ heterostructure, a structure we denote a quantum material spintronic resonator (QM-SR). First, the metal-insulator transition in ${\mathrm {V}}_2{\mathrm {O}}_3$ is shown to modify the amplitude of the ferromagnetic resonance response. Second, the structural phase transition in ${\mathrm {V}}_2{\mathrm {O}}_3$ changes the resonance frequency. Third, these effects are hysteretic and can be controlled by a dc current bias that locally heats the nanoconstriction region. The latter result demonstrates the potential of quantum materials to form current controlled spintronic resonators with memory.

## Materials and geometry

**Figure 1:**
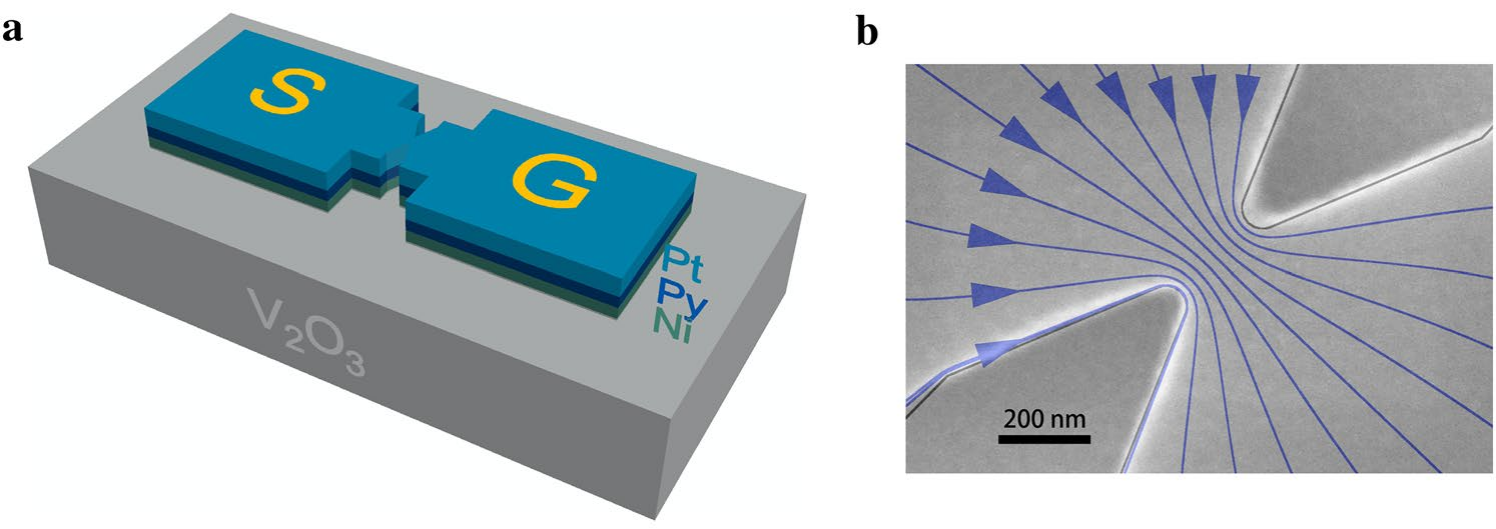
A nanoconstriction spintronic resonator. (**a**) Schematic shows the layers and the geometry. The signal (S) and ground (G) electrical contacts are indicated (not to scale). (**b**) SEM image of a 200 nm nanoconstriction. The blue lines with arrows illustrate the current flow through the constriction. The line density is proportional to the current density determined from a COMSOL simulation.

We studied $${\mathrm {V}}_2{\mathrm {O}}_3 (100\,{\hbox {nm}}) | {\mathrm {Ni}}(1.5\,{\hbox {nm}})| {\mathrm {Ni}}_{80} {\mathrm {Fe}}_{20}(1.5\,{\hbox {nm}})| {\mathrm {Pt}}(5\,{\hbox {nm}})$$ thin films on sapphire substrates. $${\mathrm {V}}_2{\mathrm {O}}_3$$ has been shown to strongly influence the properties of Ni, particularly its coercive field and ferromagnetic resonance response^3,5^. This occurs at $${\mathrm {V}}_2{\mathrm {O}}_3$$’s structural phase transition and is associated with the large magnetostriction coefficient of Ni. For this reason our structure incorporates a $${\mathrm {V}}_2{\mathrm {O}}_3$$$$|\hbox {Ni}$$ interface. The Ni layer is exchange coupled to the Permalloy (Py, $${\hbox {Ni}}_{80}{\hbox {Fe}}_{20}$$) layer such that the spin Hall effect in the Pt layer excites spin dynamics in the ferromagnetically coupled $${\hbox {Py}}|{\hbox {Ni}}$$ layers. Nanoconstrictions were formed using electron-beam lithography and Ar ion milling, with the transition metal layers etched to expose the $${\mathrm {V}}_2{\mathrm {O}}_3$$ surface, as illustrated schematically in Fig. 1a. The constriction’s lateral scale was varied from 50 to 200 nm and connected to signal and ground contacts, labeled “S” and “G” in Fig. 1a. A scanning electron microscopy (SEM) image of the constriction is shown in Fig. 1b. The bottom-left and top-right corner regions in the image are the surfaces of the $${\mathrm {V}}_2{\mathrm {O}}_3$$ and the region with blue lines and arrows is within the metal layers. The constriction shown is 200 nm wide and in the following we focus on the properties of spintronic resonators of this width.

Current flows from the signal (S) to ground (G) contacts through the nanoconstriction. We use finite-element simulations (COMSOL) to model the current flow when ${\mathrm {V}}_2{\mathrm {O}}_3$ is in an insulating state; the results are overlaid on the SEM image, with the density of the lines indicating the current density. As expected, the highest current densities are in the nanoconstriction region. This is thus the region that experiences the largest spin-orbit torques and the region in which spin dynamics are excited, as seen in micro-Brillouin light scattering studies of metallic samples of a similar geometry^8^.

**Figure 2:**
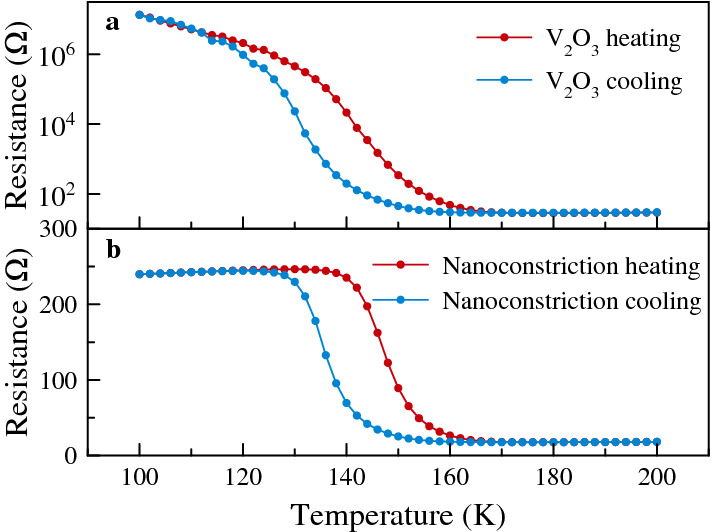
Sample electrical properties. (**a**) Resistance versus temperature measurement of a ${\mathrm {V}}_2{\mathrm {O}}_3$ film. (**b**) Resistance versus temperature measurement of a 200 nm lateral scale nanoconstriction formed from ${\mathrm {V}}_2{\mathrm {O}}_3 | {\mathrm {Ni}} | {\mathrm {Py}} | {\mathrm {Pt}}$ layers.

Figure 2 shows the electrical properties of a ${\mathrm {V}}_2{\mathrm {O}}_3$ film (Fig. 2a) and a 200 nm nanoconstriction formed in ${\mathrm {V}}_2 {\mathrm {O}}_3 | {\mathrm {Ni}} | {\mathrm {Py}} | {\mathrm {Pt}}$ layers (Fig. 2b). The ${\mathrm {V}}_2{\mathrm {O}}_3$ film’s resistance increases by more than 4 orders of magnitude as the temperature decreases from 160 to 100 K (Fig. 2a). This is characteristic of the first-order phase transition in ${\mathrm {V}}_2{\mathrm {O}}_3$ from a paramagnetic metal to an antiferromagnetic insulator, with an associated structural phase transition from a rhombohedral to a monoclinic structure^18–20^. The transition is hysteretic, with about a 10 K shift in resistance between data taken on heating and cooling the sample. We observe similar hysteresis in the magnetic properties of ${\mathrm {V}}_2 {\mathrm {O}}_3 | {\mathrm {Ni}} | {\mathrm {Py}} | {\mathrm {Pt}}$ layers (see the Supplementary Material). The nanoconstriction’s electrical characteristics are also hysteretic (Fig. 2b). At high temperature ($\gtrsim 150\,{\hbox {K}}$) its resistance is less than ${20}\,{\Omega }$, while below 140 K its resistance increases to just over ${200}\,{\Omega }$. This is consistent with parallel conduction in the metal layers and ${\mathrm {V}}_2{\mathrm {O}}_3$ at high temperature—with ${\mathrm {V}}_2{\mathrm {O}}_3$ in a metallic state—and conduction predominately in the metal layers at low temperatures, when the ${\mathrm {V}}_2{\mathrm {O}}_3$ shows insulating behavior. The nanoconstriction’s slightly decreasing resistance with decreasing temperature below the ${\mathrm {V}}_2{\mathrm {O}}_3$’s metal-insulator transition temperature is further evidence of metallic conduction.

## Nanoconstriction response

**Figure 3:**
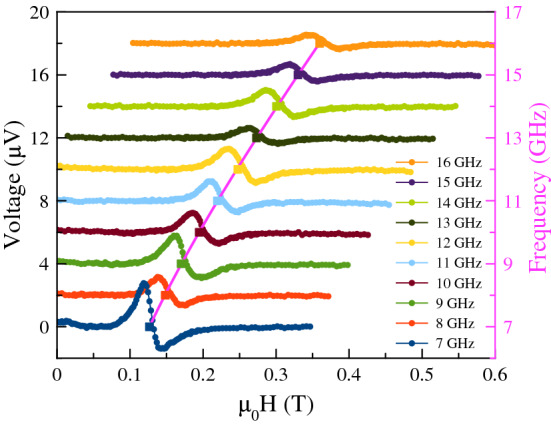
ST-FMR spectra. The field is swept at a fixed rf frequency of 7 to 16 GHz and the curves are offset from one another to align their baseline with their rf frequency on the right axis. Prior to each measurement the sample is cooled to 100 K and then raised to the measurement temperature of 120 K to ensure the ${\mathrm {V}}_2{\mathrm {O}}_3$ is in an insulating state. The square symbols indicate the resonance field associated with the rf frequency on the right hand y-axis. The pink curve is a fit to the Kittel model described in the main text.

To characterize the response of the nanoconstriction we conduct spin-transfer ferromagnetic resonance (ST-FMR) experiments, as described in the Methods section. We first measure the sample using ST-FMR at 120 K, below the metal-insulator transition temperature in ${\mathrm {V}}_2{\mathrm {O}}_3$, at which current flows mainly through the metal layers. The applied in-plane field is swept from 0.6 to 0 T at fixed rf frequency, which is varied from 7 to 16 GHz. The results are shown in Fig. 3. The curves are offset by $2\,{\upmu }{\hbox {V}}$ to make the different frequency spectra visible and align their baseline with the rf frequency on the right-hand axis. The spectra are then fit to the sum of the derivative of Lorentzian and anti-Lorentzian to determine the resonance parameters^21^. The resulting resonance field is indicated by the solid squares in the figure. A fit to the rf frequency-resonance field dispersion with the Kittel model for this geometry^22^

1 $$\begin{aligned} f = \frac{\mu _0 \gamma }{2 \pi } \sqrt{\left( H + H_{\mathrm {A}} \right) \left( H + H_{\mathrm {A}} + M_{\mathrm {eff}} \right) }, \end{aligned}$$

is shown as the pink curve in Fig. 3. Here *H* is the applied field, $H_{\mathrm {A}}$ is in-plane shape anisotropy field associated with the nanoconstriction and $M_{\mathrm {eff}}$ characterizes the easy-plane anisotropy. $\mu_0$ is the permeability of free space and $\gamma$ is the gyromagnetic ratio. The fit gives $\gamma /2\pi = 29.2(7){\hbox {GHz/T}}$, $\mu_0 H_A = -0.048(2){\hbox {T}}$ and $\mu_0 M_{\mathrm {eff}} = 0.66(5){\hbox {T}}$. The Gilbert damping can be determined from the dependence of the spectral linewidth on applied field to be $\alpha = 0.042(5)$ (see the Supplementary Materials). As only one resonance peak is observed, it is clear that the Ni and Py are strongly exchange coupled and respond collectively to the spin torques. (See the Supplementary Material for discussion of the FMR spectra and a comparison to FMR results on unpatterned films.)

**Figure 4:**
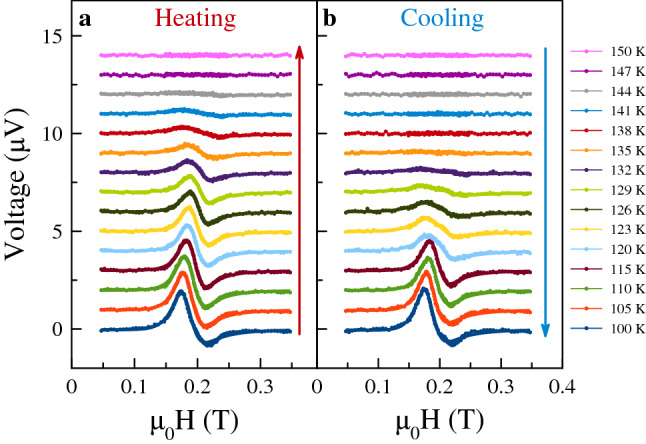
ST-FMR on heating and cooling through the ${\hbox {V}}_{2}{\hbox {O}}_{3}$ phase transition. (**a**) Data obtained on heating the sample, from 100 to 150 K. (**b**) Data obtained on cooling the sample within the same temperature range. The long red (in (**a**)) and blue (in (**b**)) arrows indicate in the sense in which the temperature was changed. In both cases the rf frequency is fixed at 10 GHz.

**Figure 5:**
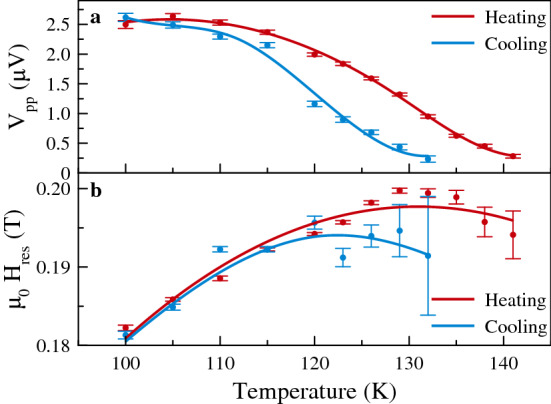
ST-FMR hysteresis. Signal amplitudes (**a**) and resonance fields (**b**) at an rf frequency of 10 GHz obtained from fits to the data in Fig. 4. The red points are from ST-FMR data taken on heating and the blue symbols from data on cooling the sample. The lines are guides to the eye. The increasing uncertainty in the resonance field with increasing temperature is associated with the decreasing signal amplitude with temperature (see Fig. 4).

When ${\mathrm {V}}_2{\mathrm {O}}_3$ undergoes its metal-insulator and structural phase transitions, a change of the ferromagnetic resonance response is expected. Figure 4 shows ST-FMR spectra with varying temperature at fixed rf frequency $f = {10}\,{\hbox {GHz}}$. The curves are offset by ${1}\,{\upmu}{\hbox {V}}$ to make the trends visible. Further, the data are taken on heating (Fig. 4a) and cooling the sample (Fig. 4b). The results at the highest (150 K) and lowest temperature (100 K) are similar in both cases. But near the ${\mathrm {V}}_2{\mathrm {O}}_3$ metal-insulator transition (e.g. $\simeq {130}\,{\hbox {K}}$) they differ significantly. Foremost, the amplitude of the response depends on the sample’s prior history; whether the ${\mathrm {V}}_2{\mathrm {O}}_3$ was in an insulating or metallic state prior to the measurement.

The amplitude of the response is plotted versus temperature in Fig. 5a. It indeed shows a strong hysteresis. Starting at 100 K the amplitude decreases monotonically with increasing temperature. At 150 K—when the ${\mathrm {V}}_2{\mathrm {O}}_3$ is in a metallic state—the ST-FMR signal vanishes. There is thus clear hysteresis in the amplitude of the ferromagnetic resonance response that mirrors the hysteresis in the resistance of the sample (shown in Fig. 2b). Furthermore, the ferromagnetic resonance frequency is modified by the ${\mathrm {V}}_2{\mathrm {O}}_3$ phase transition. Starting at 100 K the resonance field increases with increasing temperature reaching a maximum at about 130 K. The resonance field can differ by $\mu_0 \delta H = {50}\,{\hbox {mT}}$ between the heating and the cooling cycles. The corresponding change in the ferromagnetic resonance mode frequency at fixed field can be calculated as $\delta H \left( \mathrm{d}{f}/\mathrm{d}{H} \right)$ using Eq. (1), which is of order of 1 GHz.

This data highlights several new and important results. First, the amplitude of the resonance depends strongly on temperature as shown in Figs. 4 and 5a. This is because the spin torques are associated with current flow in the Pt layer and conduction in the ${\mathrm {V}}_2{\mathrm {O}}_3$ reduces the current flow in the Pt layer and thus the spin torques on the ferromagnetic layers. Second, the structural phase transition in ${\mathrm {V}}_2{\mathrm {O}}_3$ changes the ferromagnetic resonance condition, as shown in Fig. 5b. Third, the first order phase transition in ${\mathrm {V}}_2{\mathrm {O}}_3$ leads to a hysteretic nanoconstriction response. This is as seen from the resistance of data (Fig. 2b), the ST-FMR response on heating and cooling in Fig. 4 and the amplitude and resonance fields as a function of temperature shown in Fig. 5. These results highlight the unique characteristics of QM-SRs: their response depends on their prior history, i.e. they have memory of their prior state.

## Tuning response with a dc current

**Figure 6:**
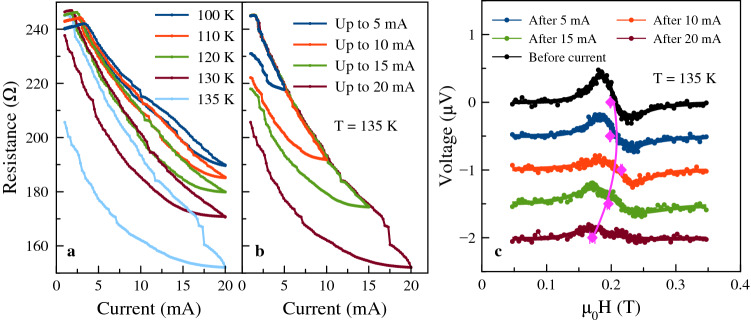
Tuning nanoconstriction characteristics with a dc current. (**a**) Nanoconstriction resistance versus current up to 20 mA starting at different initial temperatures. (**b**) Resistance versus current up to different maximum currents at a fixed temperature of 135 K. After each resistance-current sweep the sample was initialized by cooling to 100 K. (**c**) The ST-FMR spectra corresponding to the states in (**b**) at zero current, i.e. after applying the dc current indicated. Each curve is offset by ${0.5}\,{\upmu}{\hbox {V}}$. As a reference the black curve shows the ST-FMR spectra before applying current. The resonance fields are marked by the pink squares and the trend is illustrated by the pink curve.

We further show that the nanoconstriction response can be tuned locally with a dc current. The control is local because the sample heating is largest where the current density is highest, i.e. in the nanoconstriction region. Figure 6a,b show that a dc current can change the nanoconstriction’s resistance. In Fig. 6a the dc current is increased to 20 mA starting at different initial temperatures, from 100 to 135 K. At 100 K the resistance of the contact returns to its original value after increasing the current to 20 mA and then decreasing it to zero. On the other hand, at 135 K there is a memristive-like response, the contact resistance changes significantly on ramping the current up to 20 mA and back to zero.

This enables control of the resistance of the nanoconstriction at a fixed temperature as shown in Fig. 6b. At 135 K the maximum current is varied from 5 to 20 mA. In each case the sample is cooled to 100 K prior to the current ramp to set it back to the same initial resistance state. The larger the maximum applied current the lower the sample resistance at zero current. Figure 6c shows the ST-FMR spectra corresponding to the final state in Fig. 6b. Each curve is offset by ${0.5}\,{\upmu}{\hbox {V}}$ to make it easier to see the individual spectra. The dc current at fixed environmental temperature leads to a clear change in the signal amplitude (Fig. 6c). The larger the prior dc current applied, the smaller the signal, which indicates the ${\mathrm {V}}_2{\mathrm {O}}_3$ is deeper in its phase-transition region. Further, the resonance field changes as well, as shown by the pink points that mark $H_{\mathrm {res}}$ in Fig. 6c. The resonance shifts to the lower field end when ${\mathrm {V}}_2{\mathrm {O}}_3$ enters the phase transition. In addition, the resonance peak appears to split into two peaks after applying a current greater than about 15 mA. This suggests the coexistence of metallic and insulating ${\mathrm {V}}_2{\mathrm {O}}_3$ regions in the nanoconstriction region^23^, with two different strain states and resonance fields, as reported in^5^.

## Summary

These results highlight new spintronic functionalities that can be achieved by coupling of a transition metal oxide and metallic ferromagnets in a nanoconstriction geometry. Novel characteristics are associated with the metal-insulator transition in ${\mathrm {V}}_2{\mathrm {O}}_3$, which can shunt part of the current and be used to control the output signal. The ${\mathrm {V}}_2{\mathrm {O}}_3$ structural phase transition modifies the ferromagnetic resonance frequency and renders both the signal amplitude and frequency as functions of the prior sample state, i.e. provides a memory function. Further, dc current can be used to modify the nanoconstriction’s response, “in-situ” by local heating. These characteristics can be useful as spintronic resonators synapses, as discussed in^16,17^. In QM-SRs a dc current can adjust a synaptic “weight.” Finally, the effects could be enhanced by using ferromagnetic materials with larger spin-phonon coupling and magnetoelastic effects, e.g. FeGa^24^.

## Methods

### Sample fabrication

A 100 nm thick ${\mathrm {V}}_2{\mathrm {O}}_3$ film is grown by rf sputtering on an a-cut ${\mathrm {Al}}_2 {\mathrm {O}}_3$ substrate at ${750}\,^{\circ }{\hbox {C}}$ substrate temperature in 7.7 mTorr pure Ar atmosphere. After the ${\mathrm {V}}_2{\mathrm {O}}_3$ growth, the sample is thermally quenched at the rate of $\sim 100\,^{\circ }\hbox {C/min}$^25^. After the sample cools down to room temperature, a ${\mathrm {Ni}} ({1.5}\,{\hbox {nm}}) | {\mathrm {Ni}}_{80} {\mathrm {Fe}}_{20} ({1.5}\,{\hbox {nm}}) | {\mathrm {Pt}} ({5}\,{\hbox {nm}})$ stack is sputtered on top of the ${\mathrm {V}}_2{\mathrm {O}}_3$ layer without breaking vacuum. Ni and Py layers are grown by rf sputtering, and the Pt layer is deposited by dc sputtering, both in an Ar pressure of 4.1 mTorr. The nanoconstriction is made by electron-beam lithography. The film is then etched using Ar ion milling in 50 mTorr Ar pressure at 200 W power. The etching process stops at the top of the ${\mathrm {V}}_2{\mathrm {O}}_3$ surface.

### ST-FMR measurement

In our ST-FMR experiments we apply an rf current to the sample, which through spin orbit torques in the Pt layer, excite spin dynamics in the ferromagnetic layers. Ferromagnetic resonance excited by the current leads to resistance oscillations due to the anisotropic magnetoresistance of the ferromagnetic layers. The nanoconstriction’s resistance oscillates at the same rf frequency as the current leading to a dc voltage, known as the spin-diode effect^26,27^. In order to have a large resistance response, we apply an in-plane field at $45^\circ$ to the current direction. In our measurements, we fix the rf frequency and sweep the external magnetic field from high to low field, 0.6 to 0 T. Further, we modulate the field to increase the signal-to-noise ratio, with a modulation amplitude $\delta H$ much less than the resonance linewidth ($\mu_0 \delta H = {0.2}\,{\hbox {mT}}$)^21,28^.

## Supplementary Information

Supplementary Information.

## References

[1] Hellman F (2017). Interface-induced phenomena in magnetism. Rev. Mod. Phys..

[2] Saerbeck T (2014). Coupling of magnetism and structural phase transitions by interfacial strain. J. Mater. Res..

[3] De La Venta J (2014). Coercivity enhancement in $${\rm {V}}_2 {\rm {O}}_3/\text{Ni }$$ bilayers driven by nanoscale phase coexistence. Appl. Phys. Lett..

[4] Lauzier J, Sutton L, De La Venta J (2018). Magnetic irreversibility in $$\text{ VO}_2/\text{Ni }$$ bilayers. J. Phys.: Condens. Matter.

[5] Ramírez JG (2016). Collective mode splitting in hybrid heterostructures. Phys. Rev. B.

[6] Zhu M (2020). Modulation of spin dynamics across metal to insulator transitions in hybrid heterostructures. J. Mater. Res. Technol..

[7] Silva TJ, Rippard WH (2008). Developments in nano-oscillators based upon spin-transfer point-contact devices. J. Magn. Magn. Mater..

[8] Demidov V, Urazhdin S, Zholud A, Sadovnikov A, Demokritov S (2014). Nanoconstriction-based spin-Hall nano-oscillator. Appl. Phys. Lett..

[9] Hoffmann A (2013). Spin Hall effects in metals. IEEE Trans. Magn..

[10] Miron IM (2011). Perpendicular switching of a single ferromagnetic layer induced by in-plane current injection. Nature.

[11] Liu L, Moriyama T, Ralph D, Buhrman R (2011). Spin-torque ferromagnetic resonance induced by the spin Hall effect. Phys. Rev. Lett..

[12] Slavin A, Tiberkevich V (2009). Nonlinear auto-oscillator theory of microwave generation by spin-polarized current. IEEE Trans. Magn..

[13] Finocchio, G. *et al.* Perspectives on spintronic diodes. arXiv preprint arXiv:2103.13793 (2021).

[14] Torrejon J (2017). Neuromorphic computing with nanoscale spintronic oscillators. Nature.

[15] Romera M (2018). Vowel recognition with four coupled spin-torque nano-oscillators. Nature.

[16] Leroux N (2021). Radio-frequency multiply-and-accumulate operations with spintronic synapses. Phys. Rev. Appl..

[17] Leroux, N. *et al.* Hardware realization of the multiply and accumulate operation on radio-frequency signals with magnetic tunnel junctions. arXiv preprint arXiv:2103.11993 (2021).

[18] Dernier PD, Marezio M (1970). Crystal structure of the low-temperature antiferromagnetic phase of $${{\rm V}}_2 {{\rm O}}_3$$. Phys. Rev. B.

[19] Hansmann P (2013). Mott-Hubbard transition in $${{\rm V}}_2 {{\rm O}}_3$$ revisited. Physica Status Solidi (b).

[20] Brockman JS (2014). Subnanosecond incubation times for electric-field-induced metallization of a correlated electron oxide. Nat. Nanotechnol..

[21] Xu J-W, Kent AD (2020). Charge-to-spin conversion efficiency in ferromagnetic nanowires by spin torque ferromagnetic resonance: reconciling lineshape and linewidth analysis methods. Phys. Rev. Appl..

[22] Farle M (1998). Ferromagnetic resonance of ultrathin metallic layers. Rep. Prog. Phys..

[23] Chen X-B, Shin J-H, Kim H-T, Lim Y-S (2012). Raman analyses of co-phasing and hysteresis behaviors in $${{\rm V}}_2 {{\rm O}}_3$$ thin film. J. Raman Spectrosc..

[24] Godejohann F (2020). Magnon polaron formed by selectively coupled coherent magnon and phonon modes of a surface patterned ferromagnet. Phys. Rev. B.

[25] Trastoy J, Kalcheim Y, del Valle J, Valmianski I, Schuller IK (2018). Enhanced metal-insulator transition in $${{\rm V}}_2 {{\rm O}}_3$$ by thermal quenching after growth. J. Mater. Sci..

[26] Tulapurkar AA (2005). Spin-torque diode effect in magnetic tunnel junctions. Nature.

[27] Sankey JC (2006). Spin-transfer-driven ferromagnetic resonance of individual nanomagnets. Phys. Rev. Lett..

[28] Gonçalves A (2013). Spin torque ferromagnetic resonance with magnetic field modulation. Appl. Phys. Lett..

