# Supplementary material for: A quantum material spintronic resonator
Source: Sci Rep. 2021 Jul 23;11:15082. doi: 10.1038/s41598-021-93404-4 (PMC8302732; doi:10.1038/s41598-021-93404-4)
Supplement: Supplementary file 1 — Supplementary Information. [file 41598_2021_93404_MOESM1_ESM.pdf]

A Quantum Material Spintronic Resonator
(Supplementary Materials)

Jun-Wen Xu,[1,*] Yizhang Chen,[1] Nicolás M. Vargas,[2] Pavel Salev,[2] Pavel N. Lapa,[2] Juan Trastoy,[3] Julie Grollier,[3] Ivan K. Schuller,[2] and Andrew D. Kent[1]

[1]*Center for Quantum Phenomena, Department of Physics, New York University, New York 10003, USA*

[2]*Center for Advanced Nanoscience, Department of Physics, University of California-San Diego, La Jolla, CA, USA*

[3]*Unité Mixte de Physique, CNRS, Thales, Université Paris-Saclay, Palaiseau, France*

(Dated: July 12, 2021)

* junwen.xu@nyu.edu

I. COERCIVE FIELD VS. TEMPERATURE

In order to characterize the temperature dependence of the magnetic properties of the films we used magneto-optical Kerr effect polarimetry (MOKE) and vector network analyzer-ferromagnetic resonance (VNA-FMR). The layer stack of the film is $V_2O_3(100\,\mathrm{nm})|\mathrm{Ni}(10\,\mathrm{nm})|$ $\mathrm{Ni}_{80}\mathrm{Fe}_{20}(10\,\mathrm{nm})|\mathrm{Pt}(3\,\mathrm{nm})$. Having a film sample with a thicker ferromagnetic layer increases the signal-to-noise ratio—particularly in the FMR measurements—and gives the temperature dependence of the material properties of interest for our study.

Figure S1(a) shows the temperature dependence of the film's coercivity $\mu_0 H_\mathrm{c}$ obtained by MOKE during both cooling and heating cycles. The structural phase transition in V_2O_3 induces strain in the Ni layer and, due to the inverse magnetoelastic effect, there is an enhancement of the coercivity. The hysteresis is associated with the coexistence of V_2O_3 phases during the transition [1].

II. EFFECTIVE MAGNETIZATION

Figure S1(b) shows the FMR resonance field at 13 GHz in the field perpendicular configuration as a function of temperature. We also find that there is a hysteresis between the cooling and heating processes, which mirror the hysteresis in the V_2O_3 film's and nanoconstriction's resistance shown in Fig. 2 in the main text.

What is more, we find that before the structural phase transition of V_2O_3, the resonance field decreases with temperature, and it starts to increase after the transition temperature, which seems contradictory to the results of the ST-FMR study shown in Fig. 5(b), but this is not the case. We associate the change of the resonance field with the change in the film's effective magnetization at the phase transition. On one hand, for the ST-FMR measurements, the magnetic field is applied in the plane of the sample, so the dispersion curve follows the in-plane-field Kittel model,

$$f = \frac{\mu_0 \gamma}{2\pi} \sqrt{\left(H + H_\mathrm{A}\right)\left(H + H_\mathrm{A} + M_\mathrm{eff}\right)}\,. \tag{1}$$

On the other hand, for the film FMR measurement, the magnetic field is perpendicular to the film, so the dispersion curve follows the out-of-plane-field Kittel model,

$$f = \frac{\mu_0 \gamma}{2\pi} \left(H - M_\mathrm{eff}\right)\,. \tag{2}$$

Thus for the same trend of the changes in the effective magnetization versus temperature, the corresponding resonance fields at a fixed frequency changes in the opposite direction.

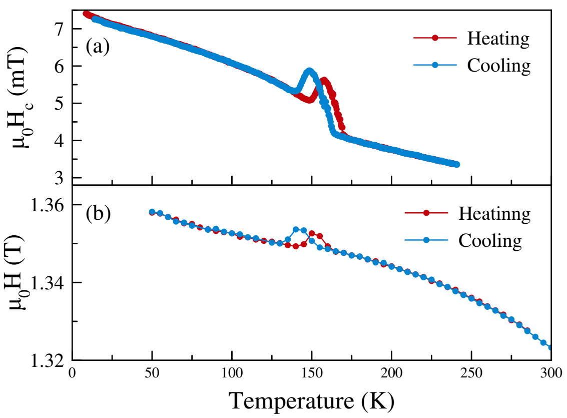

Figure S1. (a) Coercivity measured by MOKE with the applied field in the film plane versus temperature on heating and cooling the sample. (b) Resonance field measured by FMR at 13 GHz with the applied field perpendicular to the film plane as a function of temperature on cooling and heating the sample.

III. GILBERT DAMPING

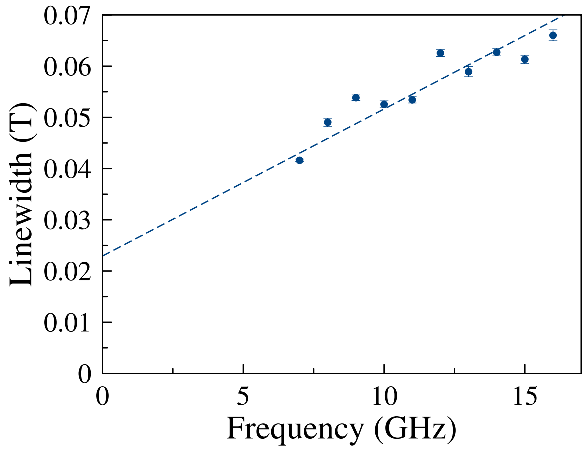

Figure S2. Linewidth of the ST-FMR spectra versus frequency at 120 K.

The linewidth $\mu_0 \Delta H$ (full width at maximum) can be found from fitting the ST-FMR spectra in Fig. 3 in the main text. The Gilbert damping α can be deduced by fitting the

linewidth data to the equation

$$\mu_0 \Delta H = \frac{4\pi\alpha f}{\gamma} + \mu_0 \Delta H_0 \,, \tag{3}$$

where γ is the gyromagnetic ratio and $\mu_0 \Delta H_0$ is the inhomogeneous linewidth broadening. The Gilbert damping calculated by fitting is $\alpha = 0.042(5)$. The damping is high relative to thick Ni and Py films, both because the ferromagnetic layers are very thin and because spin pumping into the adjacent Pt layer increases the damping.

[1] Lauzier, J., Sutton, L. & De La Venta, J. Magnetic irreversibility in VO_2/Ni bilayers. *Journal of Physics: Condensed Matter* **30**, 374004 (2018).
